# In silico analysis of nitrilase-3 protein from *Corynebacterium glutamicum* for bioremediation of nitrile herbicides

**DOI:** 10.1186/s43141-022-00332-5

**Published:** 2022-03-29

**Authors:** M. Amrutha, K. Madhavan Nampoothiri

**Affiliations:** 1 Microbial Processes and Technology Division (MPTD), CSIR-National Institute for Interdisciplinary Science and Technology (NIIST), Trivandrum, Kerala 695019 India; 2 Academy of Scientific and Innovative Research (AcSIR), Ghaziabad, 201002 India

**Keywords:** *Corynebacterium glutamicum*, Herbicides, Homology modeling, Molecular docking, Nitrilase

## Abstract

**Background:**

The nitrile compounds are produced either naturally or synthetically and are highly used in many manufacturing industries such as pharmaceuticals, pesticides, chemicals, and polymers. However, the extensive use and accumulation of these nitrile compounds have caused severe environmental pollution. Nitrilated herbicides are one such toxic substance that will persist in the soil for a long time. Therefore, effective measures must be taken to avoid its pollution to the environment. A variety of nitrile-converting bacterial species have the ability to convert these toxic substances into less toxic ones by using enzymatic processes. Among the bacterial groups, actinobacteria family members show good degradation capacity on these pollutants. The soil-dwelling Gram-positive industrial microbe *Corynebacterium glutamicum* is one such family member and its nitrile-degradation pathway is not well studied yet. In order to understand the effectiveness of using *C. glutamicum* for the degradation of such nitrile herbicides, an in silico approach has been done. In this perspective, this work focus on the structural analysis and molecular docking studies *of C. glutamicum* with nitrilated herbicides such as dichlobenil, bromoxynil, and chloroxynil.

**Results:**

The bioinformatics analysis using different tools and software helped to confirm that the genome of *C. glutamicum* ATCC 13032 species have genes (cg 3093) codes for carbon-nitrogen hydrolase enzyme, which specifically act on non-peptide bond present in the nitrile compounds. The conserved domain analysis indicated that this protein sequence was nitrilase-3 and comes under the nitrilase superfamily. The multiple sequence alignment analysis confirmed that the conserved catalytic triad residues were 40E, 115K, and 151C, and the existence of nitrilase-3 protein in the genome of *Corynebacterium* sp. was evaluated by a phylogenetic tree. The analysis of physico-chemical properties revealed that alanine is the most abounded amino acid (10.20%) in the nitrilase-3 protein, and these properties influence the substrate specificity of aliphatic and aromatic nitrile compounds. The homology modelled protein showed better affinity towards nitrile herbicides such as 2,6-dichlorobenzamide (BAM) and 3,5-dichloro-4-hydroxy-benzamide (CIAM) with the affinity value of − 5.8 and − 5.7 kcal/mol respectively.

**Conclusions:**

The in silico studies manifested that *C. glutamicum* ATCC 13032 is one of the promising strains for the bioremediation of nitrilated herbicides contaminated soil.

## Background

Nitriles are cyanide-substituted organic compounds that are synthesized on a large scale as polymers, solvents, extractants, herbicides, pharmaceuticals, etc. Similarly, it can be used as starting materials for many other industrially important chemicals [1]. Among the various nitriles, benzonitrile and its derivatives are the active ingredients in many herbicides such as dichlobenil (2, 6 dichlorobenzonitrile; commercial name, Casoron), bromoxynil (3, 5-dibromo- 4 hydroxybenzonitrile; commercial name, Buctril), and chloroxynil (3, 5-dichloro-4-hydroxybenzonitrile) [2]. These compounds are widely used in agriculture for crops such as rice, wheat, barley, corn, and berries [3]. The increased usage of nitrile compounds produces a large number of toxic effluents to the environment which causes severe health hazards in humans including gastric problems, bronchial irritation, convulsions, vomiting (nausea), respiratory distress, coma, and osteolathyrism, which results in lameness and skeletal deformities [4]. Therefore; considerable attention has been needed for the bioremediation processes to remove the pollutants from the ecosystems.

For the detoxification of toxic nitriles from environmental waste, biological methods are preferred over chemical methods, due to their eco-friendly, efficient, and cost-effective nature [5, 6]. Generally, the living organisms, predominantly microorganisms, help to degrade the environmental contaminants into less toxic forms. Various microorganisms have the ability to degrade nitrile compounds by mainly two pathways [7]. One is using nitrilase (EC 3.5.5.1) enzyme which can directly convert nitrile group into its corresponding carboxylic acid and ammonia. The second pathway is mediated by two enzymes first nitrile hydratase (EC 4.2.1.84) which converts nitrile to amide form then an amidase (EC 3.5.1.4) enzyme act on it and converts into the corresponding acid [8]. Nitrilases are widely distributed in nature and their existences are reported in plants, fungi, and more frequent in bacteria. They are mainly involved in the metabolism of various natural and synthetic nitriles. Many different bacteria include *Klebsiella*, *Acinetobacter*, *Nocardia*, *Rhodococcus*, *Pseudomonas*, *Corynebacterium*, *Arthrobacter*, etc. is known to utilize nitriles as sole sources of carbon and nitrogen [9].

The demands of nitrilase enzymes were increased tremendously because they act as good biocatalysts in different synthetic applications. Nowadays, various bioinformatics tools and techniques are preferred over conventional methods to screen or identify the novel nitrilase gene, which also helps to determine the suitable substrates that specifically bind to the protein. The available whole-genome sequence data of microbial strains open up enormous opportunities to identify the novel genes and enzymes responsible for the degradation of nitrile compounds. The *C. glutamicum* is a non-pathogenic industrially important microbe which well known for the large-scale production of amino acids. This soil-dwelling microbe also showed efficiency in the bioremediation of arsenic-contaminated areas. In the present study, we searched for a substrate-specific nitrilase gene from *C. glutamicum* ATCC 13032, which is capable of degrading nitrilated herbicides. In silico analyses were carried out to find out the gene specifically binds to the nitrilated herbicides.

## Methods

An in silico screening is used to identify any nitrilases gene from the DNA sequences of *Corynebacterium glutamicum* ATCC 13032. The protein sequence was retrieved from NCBI (https://www.ncbi.nlm.nih.gov/) [10] protein sequence database on 9 January 2020 for further analysis.

### Multiple sequence alignment analysis (MSA)

BLASTp (https://blast.ncbi.nlm.nih.gov/Blast.cgi?PAGE=Proteins) [11] was performed to identify the similar sequence present in microorganisms. The raw sequence in the fasta format was subjected to BLASTp against protein reference sequences in the NCBI database. The nine sequences were selected based on the percentage of identity showing with the query sequence. These sequences were aligned by Clustal Omega (https://www.ebi.ac.uk/Tools/msa/clustalo/) [12].

### Phylogenetic analysis

The evolutionary history of the target sequence (cg3093) was generated using the Neighbour-Joining method of MEGA (Molecular Evolutionary Genetics Analysis) X version (https://www.megasoftware.net/) [13]. The protein sequences can be used to generate the phylogenetic tree.

### Domain and motif analysis

Probable functional motifs can be identified by three different protein annotation databases, which include NCBI-CDD (Conserved Domains Database) (https://www.ncbi.nlm.nih.gov/Structure/cdd/wrpsb.cgi) [14], Pfam (Proteins Families Database) [15], and InterProScan (https://www.ebi.ac.uk/interpro/search/sequence/) [16]. The conserved domains, catalytic triad, and active site residues were predicted using NCBI-CDD. The Pfam database provides a collection of curetted and sequence aligned information of protein families. InterProScan produces high-level structure-based classification, prediction of the domain and homologous superfamily, etc. It also provides Gene Ontology (GO) terms of the respective proteins.

### Physicochemical properties of Nit-3 enzyme

The Expasy’s ProtParam (https://web.expasy.org/protparam/) [17] prediction server was used to analyze the physicochemical properties of the protein. Several parameters such as number of amino acids, molecular mass [Da], theoretical pI, number of negatively charged residues, extinction coefficients, instability index, aliphatic index, and grand average of hydropathicity (GRAVY) were computed from the FASTA sequences of the query protein.

### 2D structure prediction

PsiPred (Predict Secondary Structure) (http://bioinf.cs.ucl.ac.uk/psipred/) [18] server is an online tool used to predict the secondary structure (beta sheets, alpha helices, and coils) of the proteins from the primary sequence.

### Homology modeling

The three-dimensional structure of the putative nitrilase-3 was predicted using SWISS-MODEL [19] and accessible via the Expasy web server. The server build model from the primary amino acid sequence and model building is mainly carried out by different steps like template structure identification, alignment of the target sequence and template structure, model building and energy minimization, and finally model quality evaluation.

### Validation of the generated model

The homology modeled structure can be validated using PROCHECK (programs to check the Stereochemical Quality of Protein Structures) (https://www.ebi.ac.uk/thornton-srv/software/PROCHECK/) [20] and this software checks the stereochemical quality of a protein structure. The Ramachandran plot of predicted three-dimensional structures can be provided by analyzing the protein PDB coordinates.

### Molecular docking analysis

Molecular docking experiments were performed on AutoDock vina v1.2 [21]. It significantly provides the accurate binding mode of the ligand with respect to the protein. The different substrates (nitrile compounds and respective amides) structure was retrieved from the PubChem database [22] in the 3D sdf file format. The online software SMILES Translator (https://cactus.nci.nih.gov/translate/) was used to generate pdb files of ligand molecules. PDBQT (Protein Data Bank, Partial Charge (Q), & Atom Type (T)) structure format created using MGL (Multiple Grenade Launcher) tools. Default parameters were used for the present study.

## Results

### Sequence retrieval from NCBI database

The whole-genome sequence of *C. glutamicum* ATCC 13032 was available in the NCBI database. The carbon-nitrogen hydrolase protein (Accession: CAF20814.1) was identified and confirmed by different *in-silico* tools. The identified gene (cg3093) consists of 266 amino acids in length and it comes under the nitrilase superfamily. The respective protein sequence (FASTA format) was retrieved from the NCBI database for further analysis.

### Multiple sequence alignment and phylogenetic analysis

**Fig. 1:**
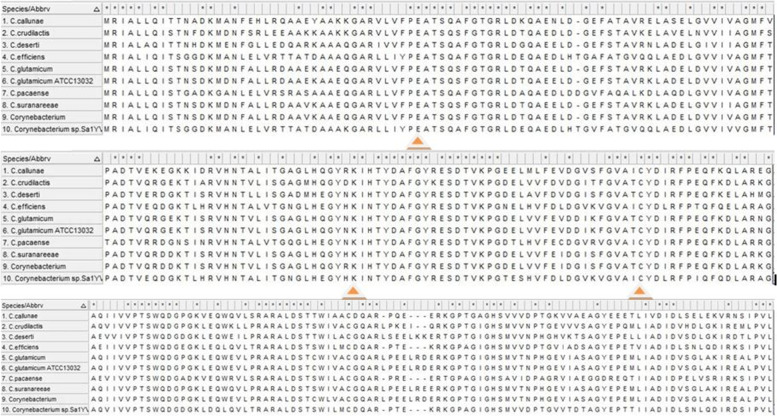
Multiple sequence alignment of nitrilase sequence of *Corynebacterium* sp. Catalytic triad residues (40E, 115K, and 151C) are highlighted in yellow color

**Fig. 2:**
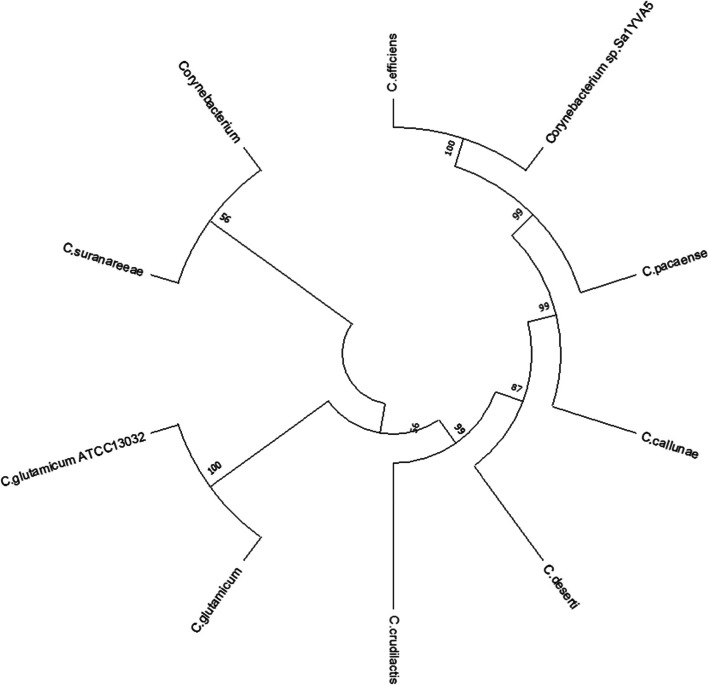
Phylogenetic tree of nitrilase protein sequences from *Corynebacterium* sp. Constructed by NJ method

The target protein sequence was blasted against the protein reference sequence database of NCBI. Based on the maximum identity score and E value, the nine sequences were selected (Table 1). For selecting the sequence, the identity values were set in the range of 65% to 100%. The *E* values also considered, in which low *E* values were selected. These sequences along with the query sequence were aligned using Clustal Omega (Fig. 1). The results obtained from MSA show that the conserved regions of the target sequence (40E, 115K, and 151C) were similar and aligned well with template sequences and hence it helps to predict the probable structure and function of the target gene from the template sequences. The evolutionary history analysis of the target gene of *C. glutamicum* ATCC 13032 was generated using the Neighbour-Joining method [23]. The bootstrap consensus tree was deduced from 1000 replicates. The evolutionary distances were computed by the Poisson correction method [24] and it is calculated as in the units of the number of amino acid substitutions per site. This analysis involved ten amino acid sequences of *Corynebacterium* sp. including the query sequence. The pairwise deletion option for all ambiguous positions in each sequence was removed and a total of 267 positions in the final dataset were generated. The sum of the branch length was 0.954 and it indicated the evolutionary time between two nodes. The analysis was conducted in MEGA X and which revealed the existence of the carbon-nitrogen hydrolase gene within the *Corynebacterium* sp (Fig. 2).

**Table 1:** The list of protein sequences selected for MSA

Sl no.	Description	Max score	Query coverage	***E*** value	Per. identity	Accession no.
**1**	*Corynebacterium glutamicum*	547	100%	0.0	100.00%	WP_011015383.1
**2**	*Corynebacterium*	527	100%	0.0	96.24%	WP_003862811.1
**3**	*Corynebacterium suranareeae*	519	100%	0.0	94.74%	WP_096458694.1
**4**	*Corynebacterium crudilactis*	459	100%	7e-162	84.59%	WP_066568400.1
**5**	*Corynebacterium deserti*	451	100%	7e-159	80.08%	WP_053545745.1
**6**	*Corynebacterium callunae*	412	100%	1e-143	75.56%	WP_015652203.1
**7**	*Corynebacterium pacaense*	364	100%	1e-124	67.79%	WP_080796956.1
**8**	*Corynebacterium efficiens*	364	100%	2e-124	66.67%	WP_035109299.1
**9**	*Corynebacterium* sp. *Sa1YVA5*	355	100%	4e-121	65.54%	WP_191733373.1

### Domain and motif analysis

The analysis of InterPro Scan database results reveals that the protein has a C-N-Hydrolase domain in their sequence from 1 to 260 amino acids. The Pfam and PROSITE profiles result also supported the above data. The conserved site of 147–167 amino acid sequence shows that it belongs to the nitrilase superfamily. The Conserved Domain Database reveals that the identified protein is nitrilase-3 and the catalytic triad residues include 40E, 115K, and 151C. Among the three catalytic residues, Cys is most conserved and play important role in enzymatic activity [25]. Gene Ontology mainly concentrates on the function of the genes and gene products and it covers three domains such as cellular component, molecular function, and biological process. The predicted biological process of nitrilase-3 was the nitrogen compound metabolic process (GO: 0006807).

### Amino acid composition and physicochemical properties of nitrilase-3

**Fig. 3:**
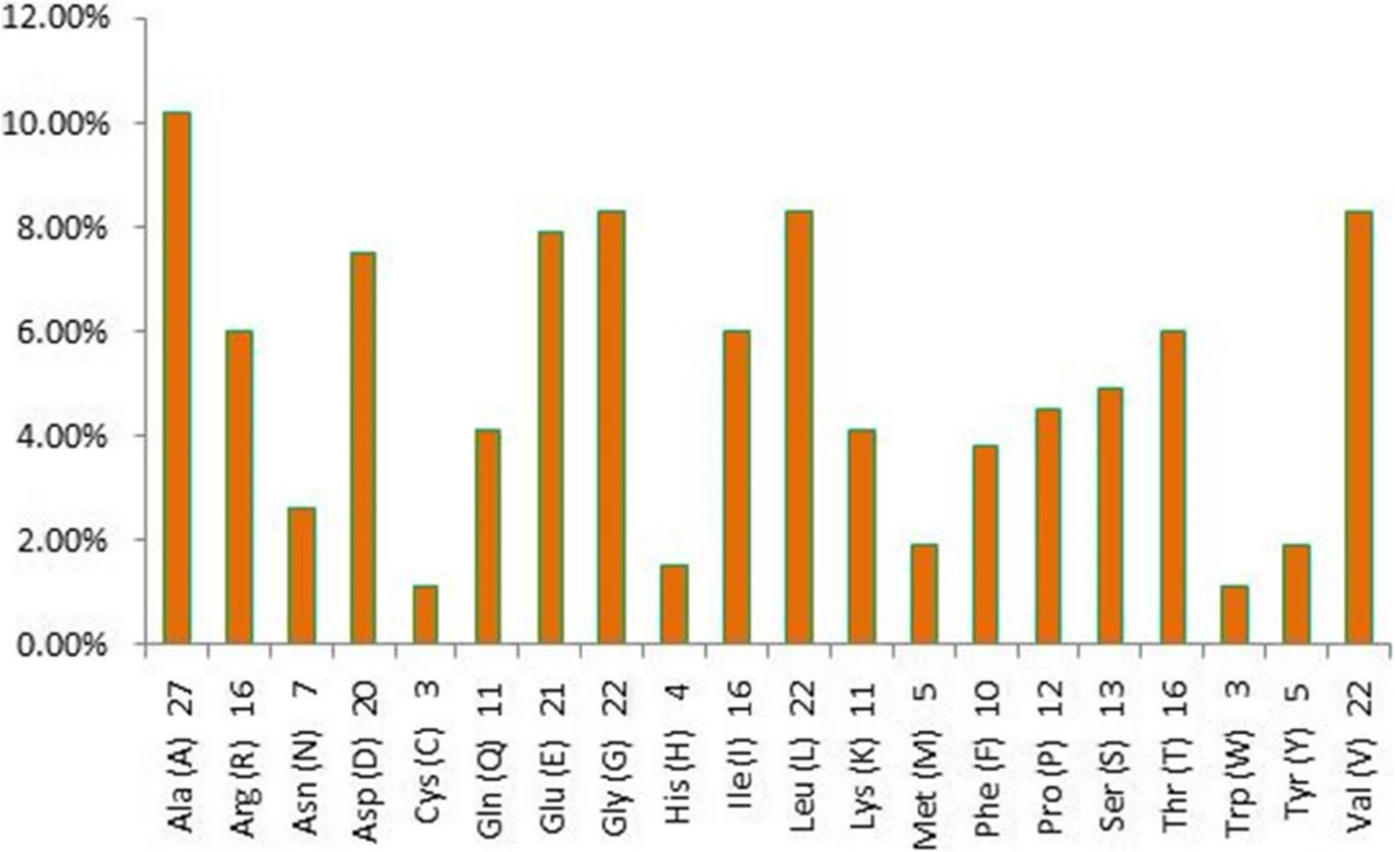
Graphical representation amino acid composition of nitrilase-3 protein

PEPSTATS analysis tool [26, 27] provided the amino acid composition of the target protein and the results show that Nit-3 protein has 266 amino acid residues in their primary sequence. Figure 3 indicated that the most abundant amino acid in Nit-3 is alanine with 10.20%, followed by glycine, leucine, and valine with the percentage of 8.30% for each of them. Tryptophan and cysteine contributed the lowest abundance residues with 1.10%. Different physical and chemical properties of the target protein sequence were computed using the ProtParam tool and the result was compared with known data of aliphatic and aromatic nitrilases (Table 2). The analysis revealed that numbers of negatively charged residues (aspartic acid 7.50% and glutamic acids 7.90%) were higher in Nit-3 protein than the positively charged residues (arginine 6.00% and lysine 4.10%). The isoelectric point is 4.76 and it states that the protein is acidic in nature. The total number of atoms is around 4079, out of that 1282 carbon (C), 2034 hydrogen (H), 354 nitrogen (N), 401 oxygen (O), 8 sulfur (S), respectively. The generated aliphatic index (Ai) was 89.85 and it evaluates the relative volume of the protein occupied by the aliphatic side chains. The protein contains a relative amount of hydrophobic amino acids is and thermally stable in nature. It also has a negative grand average hydropathicity (GRAVY) value of − 0.193 which meant that the protein is hydrophilic in nature. The instability index value helps to predict in vivo stability of a protein from its primary sequence. If the protein has an instability index value less than 40 then to be considered as stable and this protein has 21.25 suggested that protein can remain stable within a solution. The extinction coefficient measures the absorbance of a protein sample at 280 nm wavelength and the amino acid residue Cys is considered to be an important parameter in the calculation of extinction co-efficient of protein. The Estimated half-life of the protein is also determined from amino acid sequences.

**Table 2:** Comparison of physicochemical parameters of nitrilase-3 protein with aliphatic and aromatic nitrilase

Sl no.	Properties	Average value for aliphatic	Average value for aromatic	Nitrilas-3
**1**	Number of amino acids:	330-382	300-330	266
**2**	Molecular weight (Da)	38,274.0	33,693.5	29,078.86
**3**	Theoretical pI:	5.5	5.5	4.76
**4**	number of negatively charged residues (Asp + Glu):	41.7	35.8	41
**5**	number of positively charged residues (Arg + Lys):	30.3	29.2	27
**6**	Aliphatic index:	89.40	89.90	89.85
**7**	Grand average of hydropathicity (GRAVY)	00.10	00.01	-0.193
**8**	Instability index:	41.2	38.5	21.25
**9**	Extinction coefficient (M^−1^ cm^−1^) at 280 nm	50213.3	43975.0	24075
**10**	Alanine content (%)	13	9.5	10.20

### 2D structure prediction and protein localization analysis

**Fig. 4:**
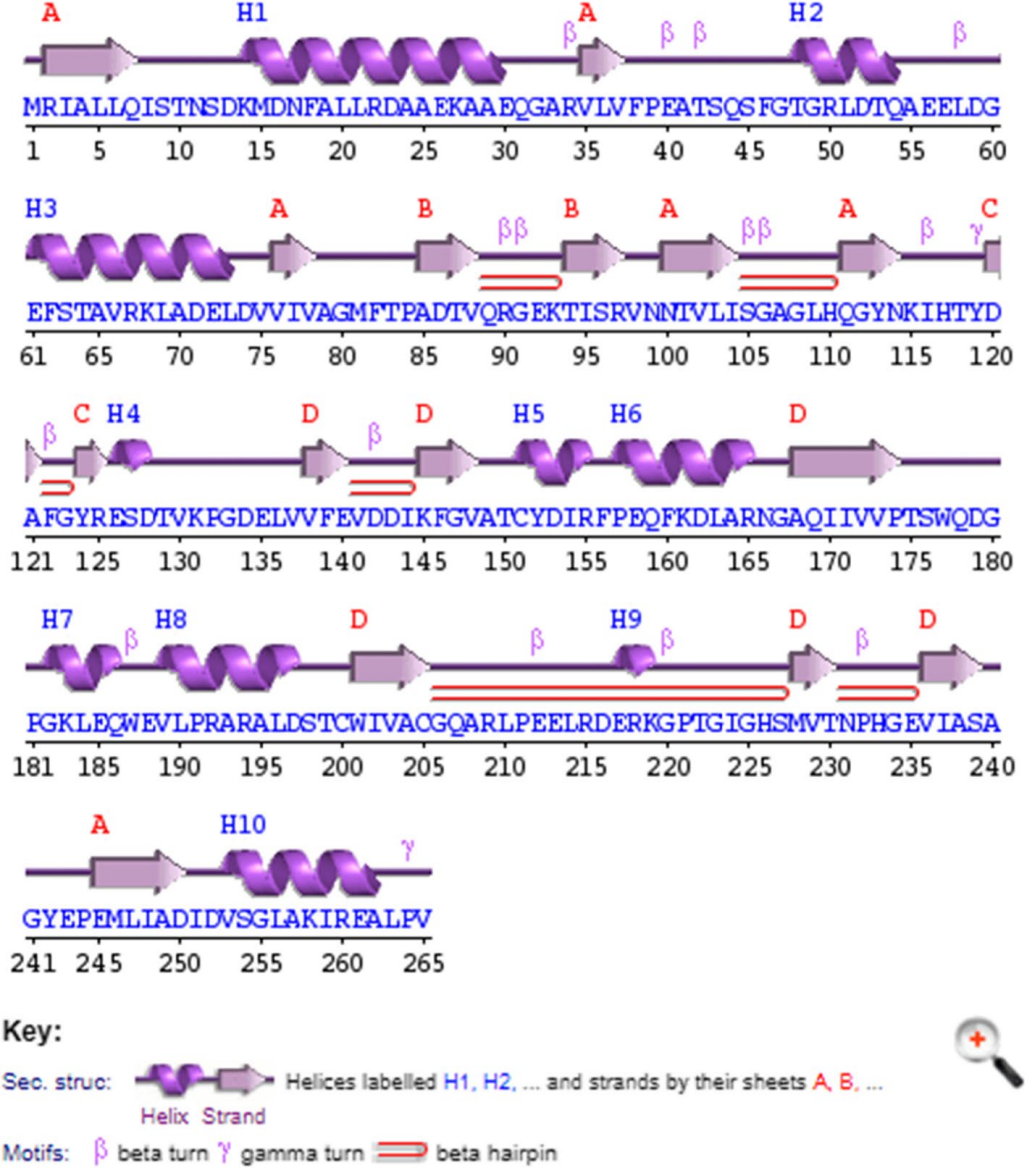
Graphical representation of the predicted secondary structures of nitrilase-3

The results obtained from PSIPRED version 4.0 show that the secondary structure of Nit-3 protein contains 10 α-helices, 16 β-strands, and 23 coils (Fig. 4). The number of coils and β-strands are high in the predicted protein than the α-helices which may indicate that these proteins are intracellular in nature. TMHMM (Transmembrane Helices Hidden Markov Models) [28] results also supported that; the protein (Nitrilase-3) does not show any transmembrane regions in it. SignalP server [29] is mostly used to find out the signal peptide regions in the proteins and Nitrilase-3 protein does not exhibit any signal peptide sequences thus, revealing its intracellular location. The protein subcellular localization was also analyzed using ProtCompB software (http://www.softberry.com/berry.phtml?topic=pcompb&group=help&subgroup=proloc), which clearly predicted that the protein is located in cytoplasmic regions with the integral score of 8.62 thus supporting the previous conclusion (Table 3).

**Table 3:** Protein localization analysis by different tools

**ProtComp**					
Location weights	LocDB	PotLocDB	NNets	Pentamers	Integral
Cytoplasmic	0.00	0.00	2.83	2.59	8.62
Membrane	0.00	0.00	0.16	0.10	0.33
Secreted	0.00	0.00	0.01	0.00	0.00
**SignalP-6.0**					
Protein type	Other	Signal peptide	Lipoprotein signal peptide	TAT signal peptide	TAT Lipoprotein signal peptide
Likelihood	1	0	0	0	0
**TMHMM- 2.0**					
Number of predicted TMHs: 0					

### Homology modelling and structure validation

**Fig. 5.**
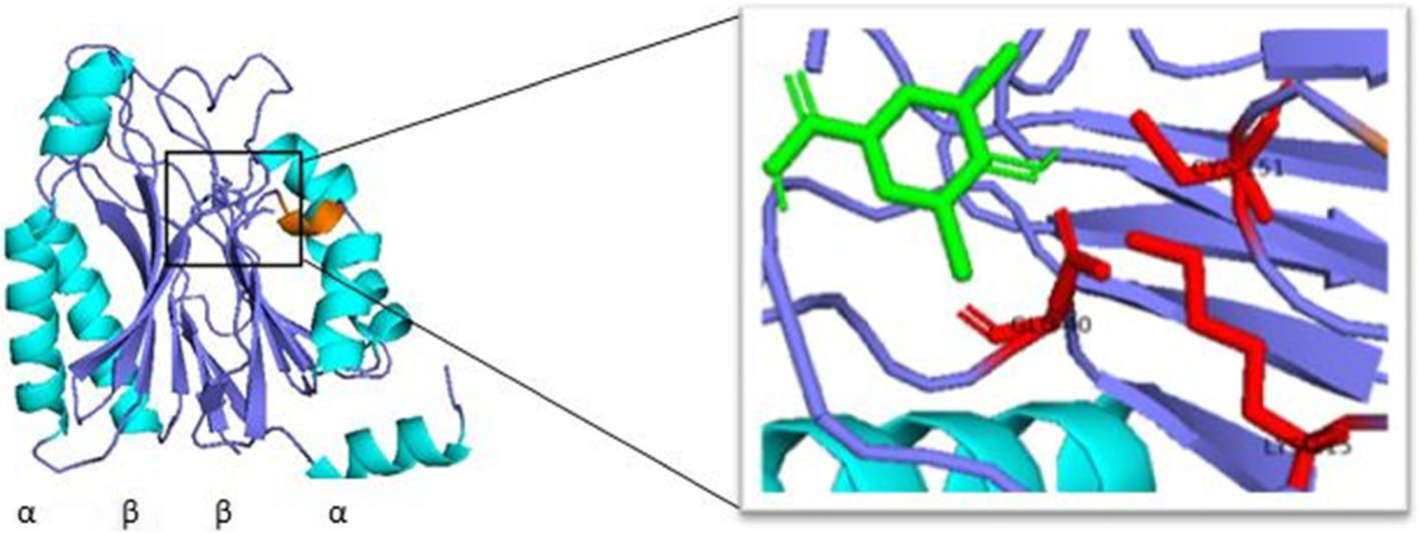
Homology modeled structure of nitrilase-3 protein: catalytic triad residues highlighted in red color, alpha helix (cyans) and beta sheets (blue) are highlighted by different colors

**Fig. 6:**
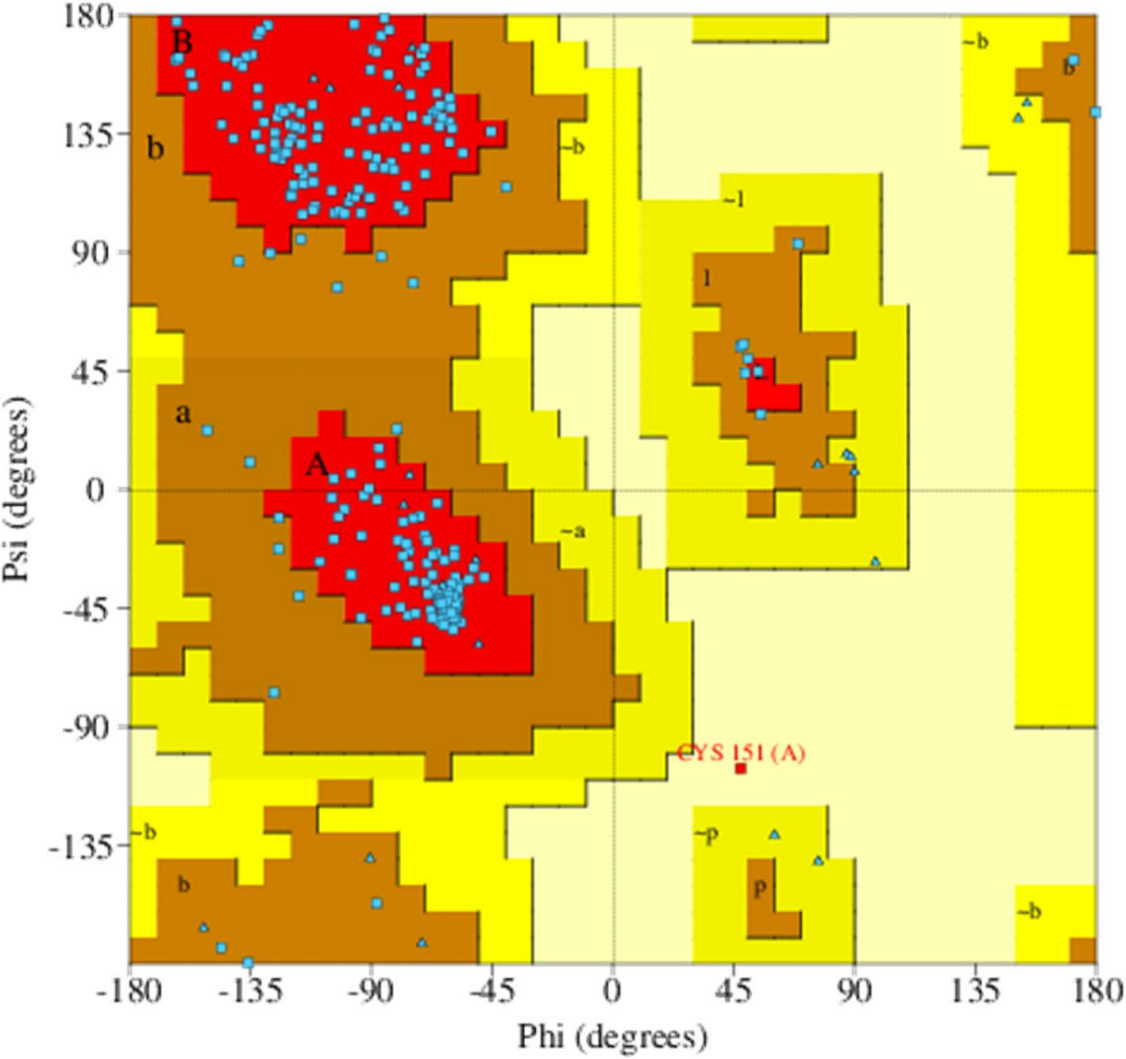
Ramachandran plot of nitrilase-3 protein by PROCHECK

The 3D structure of the Nitrilase-3 protein was generated by SWISS-MODEL accessible via the Expasy webserver (Fig. 5). The template used for the modeling of the protein is 4h5u (PDB ID). The sequence similarity and query coverage of the protein with the template were 0.35 and 0.99 respectively. The GMQE and QMEANDisCo Global value of the predicted model was 0.69 and 0.66 ± 0.05 respectively and which ensure that the modeled structure has good quality and accuracy. The quality of the modeled protein was evaluated and validated using PROCHECK, and it was primarily verified by Ramachandran plot (Fig. 6). The results were tabulated in Table 4 and confirmed that the model has 89.5% residues in the most favored regions [A, B, L], 10% residues in the additional allowed regions [a,b,l,p], no residues in the generously allowed regions [~a,~b,~l,~p] and 0.4% in the disallowed regions [XX]. Therefore, the percentage distribution of the amino acid residues suggested that the generated model was a high-quality one and normally the accuracy of the build model was dependent on the template structure.

**Table 4:** Ramachandran plot statistics

Sl no.	Model	No. of residues	% of residues
1	Residues in most favoured regions [A,B,L]	205	89.5
2	Residues in additional allowed regions [a,b,l,p]	23	10.0
3	Residues in generously allowed regions [~a,~b,~l,~p]	0	0.0
4	Residues in disallowed regions [XX]	1	0.4

### Docking analysis

**Fig. 7:**
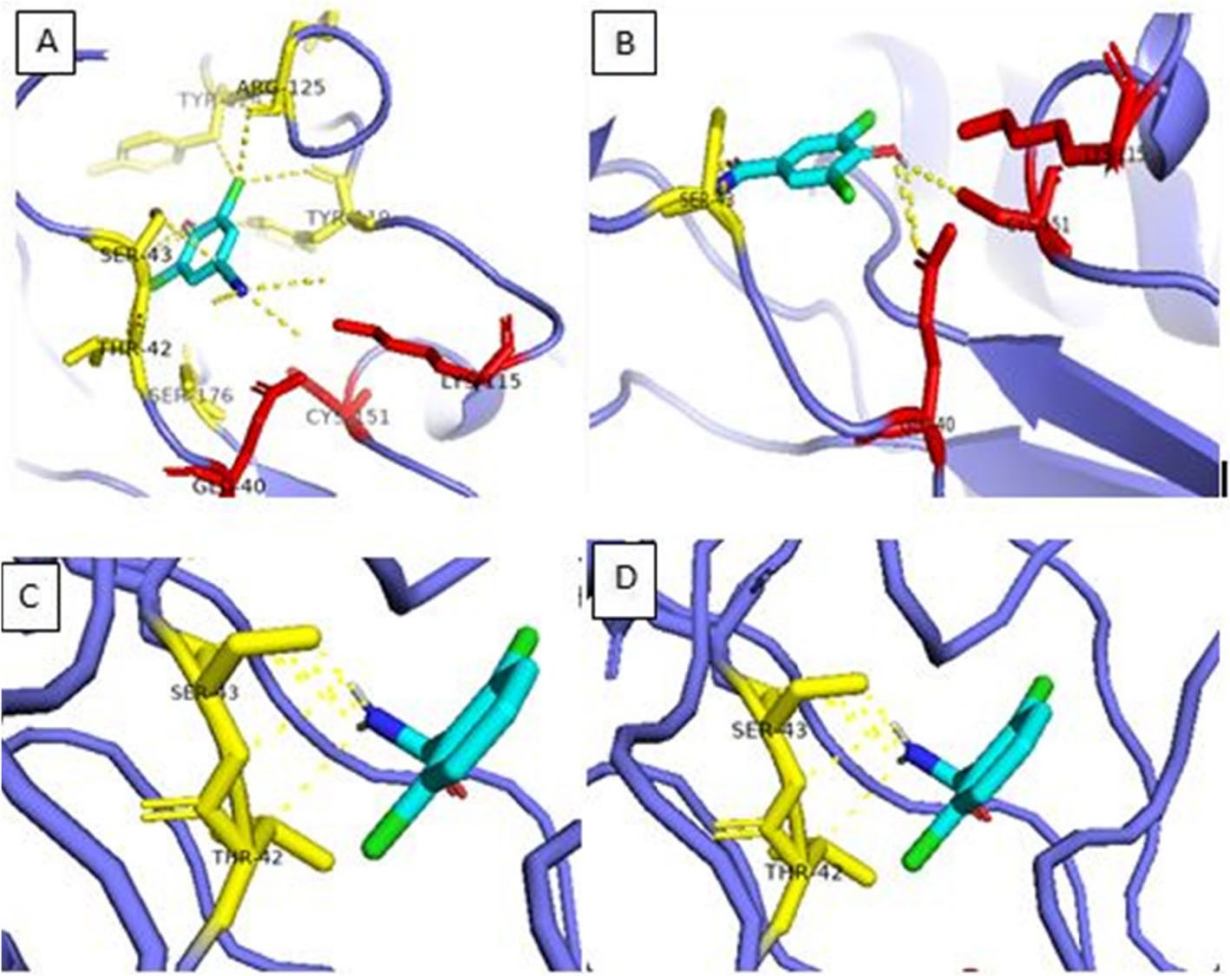
Molecular docking analysis of nitrilase-3 protein with its ligands. **A** Bromoxynil. **B** Chloroxynil. **C** BAM. **D** CIAM

The molecular docking analysis of nitrilase-3 from *Corynebacterium glutamicum* was performed to get a stable and specific substrate for the protein and also predict the protein-ligand interactions. The root mean square deviation (RMSD) < 2 was used for the protein-ligand docking. The docking result generates 9 ligands binding pose among them select the one pose which shows low RMSD. In our study, we selected the first binding pose which shows 0 RMSD value that indicates a true binding pose. Nitrilated herbicides such as dichlobenil, bromoxynil, chloroxynil, and their amide forms include 2, 6-dichlorobenzamide (BAM), and 3,5-dichloro-4-hydroxy-benzamide (CLAM) were specifically bound to the active site. Among them, BAM substrate shows high binding affinity − 5.8 kcal/mol with modeled protein followed by 5.7 kcal/mol for CLAM, 5.3 kcal/mol for benzamide, 5.2 kcal/mol for both chloroxynil and bromoxynil, − 5.1 kcal/mol for dichlobenil and − 4.8 kcal/mol for benzonitrile. All the protein-ligand interaction was calculated between chains within 3A^0^ of RMSD value. The BAM displayed polar interactions with Ser43 and Thr42 and CIAM showed interactions with Ser43, Lys115, and Cys151. Similarly, bromoxynil showed polar contact with Ser43, Lys115, and Glu40 and chloroxynil exhibit interactions with Ser43, Thr42, Ser176, Tyr119, Arg125, Tyr124, Lys115, and Glu40 (Fig. 7) The affinities towards different substrates will dependent on the active site residues of amino acids, however; they have catalytic triad motifs which are conserved in nature.

## Discussion

Microbial nitrilase is a well-chosen enzyme from the nitrilase superfamily to carry out many industrial applications and bioremediation processes. During the last decades usage of nitrilated herbicides has been increased and consequently, the toxic metabolites were liberated to the environment. These benzonitrile herbicides such as bromoxynil, chloroxynil, and dichlobenil were widely used in agriculture and household to control the weeds, and these compounds remain in the environment. There are reports on different bacterial strains which specifically degrade these compounds by two different pathways. The degradation mechanism of dichlobenil was studied extensively and they reported that nitrile hydratase act on this dichlobenil compound to convert it into the amide form (BAM) [30]. Still, BAM is a more resistant compound than dichlobenil and its degradation by microbes is rare [31]. In contrast, bromoxynil degradation by nitrilase was well studied using *Klebsiella pneumonia* [32] and other microbes. Many other bacterial isolates such as *Aminobacter*, *Pseudomonas*, *Rhizobium*, *Rhodococcus*, and *streptomyces* showed the aerobic degradation towards benzonitrile herbicides.

The present study focuses on the standard sequence-based annotation methods for effective identification of superfamily, conserved domains, active site residues, and assigning gene ontology (GO) terms for the target protein. Beyond the primary sequence, the structure of the protein gives an idea about the enzymatic function in-depth. Sharma et al. (2017) reported that the substrate selectivity of nitrilase enzyme primarily depends on the physicochemical behavior [33]. They differentiated the aliphatic and aromatic nitrilase based on these physicochemical properties. The amino acid Ala is one of the major residues that help to individualize nitrilase as if the Ala content is 9.5% it indicates that the enzyme is aromatic nitrilase and Ala is 13% means it is aliphatic nitrilase. The nitrilase-3 protein showed 10.20% Ala content it may be illustrated that this protein has the ability to hydrolyze both aliphatic and aromatic substrates. Interestingly, the instability index value of nitrilase-3 was very less compared to other aromatic and aliphatic nitrilases; this suggested that this enzyme was highly stable in nature. The GRAVY value constitutes the protein solubility and positive interaction with water molecules. The nitrilase-3 had a negative score for the GRAVY index (− 0.193) which indicates a good interaction between protein and water molecules. The nitrilase-3 protein from *C. glutamicum* exhibits a physicochemical range between or below the known aliphatic and aromatic nitrilases. These findings suggested that nitrilase-3 can be utilizing both aliphatic and aromatic nitriles as substrates.

The literature on structural studies reported that nitrilase superfamily members consist of 4 layers of the α-β-β-α sandwich fold (Fig. 5) with highly conserved catalytic triad residues including Glu-Lys-Cys [34]. These catalytic residues were located in the deep pocket that can be accessible from the molecular surfaces. The central β sheets are arranged in a parallel and an antiparallel configuration. The dimer interface regions were tightly packed with hydrophobic amino acid residues and two bundles of α-helices together to form the complete core structure of nitrilase. The identified nitrilase-3 protein also has the ability to degrade the nitrile compounds that can be evaluated by docking studies. Due to its broad spectrum of substrate specificity, the enzyme is useful for nitrile hydrolysis studies. So, these findings open up the necessity for further investigations on the nitrilase-3 enzyme for field application studies. It requires cloning and overexpressing this enzyme to check the efficacy of different nitrile herbicides.

## Conclusion

In literature, the number of high potential nitrilase-producing microbes was restricted to a few *Pseudomonas, Rhodococcus*, and *Alcaligenes* sp only. In this study, our aim was to identify and analyze the nitrilase gene from *C. glutamicum* ATCC 13032 which is responsible for the nitrile degradation processes. Various physiochemical properties of nitrilase-3 protein were compared with known standard values of aromatic and aliphatic nitrilase from that we concluded that nitrilase-3 protein contains 266 amino acids with molecular weight around 29 kDa and pI values below 7 (4.7) and negatively charged amino acid residues are dominant in the protein. Instability index, extinction coefficient, alanine content, and GRAVY values show significant differences with known values of aliphatic and aromatic nitrilase. It may influence the substrate specificity of both aliphatic and aromatic groups of nitriles. The secondary and tertiary structures were predicted using PSIPRED and SWISS-MODEL software and the structure was validated using Ramachandran plot. Based on the computational analysis the identified intracellular nitrilase-3 protein shows the degradation ability towards various herbicides compounds such as dichlobenil, bromoxynil, and chloroxynil, and its intermediate forms include BAM and CIAM. All these findings revealed that nitrilase from *C. glutamicum* is a potential enzyme for green nitrile hydrolysis.

## Data Availability

The first authors declare that all generated and analyzed data have been included in the article.

## Abbreviations

MSA: Multiple sequence alignment
MEGA X: Molecular Evolutionary Genetics Analysis
NCBI-CDD: Conserved Domains Database
Pfam: Proteins Families Database
PsiPred: Predict Secondary Structure
PROCHECK: Programs to Check the Stereochemical Quality of Protein Structures
PDBQT: Protein Data Bank
Q: Partial charge
T: Atom type
MGL tools: Multiple Grenade Launcher
BAM: 2, 6-dichlorobenzamide
CLAM: 3, 5-dichloro-4-hydroxy-benzamide
